# Implementing a biomarker‐enabled care pathway to accelerate identification of early‐stage Alzheimer’s disease in primary care

**DOI:** 10.1002/alz.095333

**Published:** 2025-01-09

**Authors:** Soo Borson, Rhoda Au, Anna H Chodos, Sam E Gandy, Holly Jain, Diana R. Kerwin, Jacobo Mintzer, Stephanie Monroe, Delecia Robinson, Donna M. Wilcock, Michelle M. Mielke

**Affiliations:** ^1^ Keck USC School of Medicine, Los Angeles, CA USA; ^2^ Boston University Chobanian & Avedisian School of Medicine and School of Public Health, Boston, MA USA; ^3^ University of California San Francisco School of Medicine, San Francisco, CA USA; ^4^ Icahn School of Medicine at Mount Sinai, New York, NY USA; ^5^ Brain Health Leaders Network, New York, NY USA; ^6^ Kerwin Medical Center, LLC, Dallas, TX USA; ^7^ Medical University of South Carolina, Charleston, SC USA; ^8^ UsAgainstAlzheimer’s, Washington, DC USA; ^9^ EviCore Healthcare, Bluffton, SC USA; ^10^ Indiana University School of Medicine, Stark Neurosciences Research Institute, Department of Neurology, Indianapolis, IN USA; ^11^ Wake Forest University School of Medicine, Winston‐Salem, NC USA

## Abstract

**Background:**

New blood‐based and digital biomarkers for Alzheimer’s disease (AD) make early detection possible at stages when novel, disease‐specific therapies are likely to be most effective. These approaches may offer less invasive, more cost‐effective alternatives to traditional methods such as cerebrospinal fluid (CSF) collection or positron emission tomography (PET) imaging for diagnosing and staging AD. Building care pathways leveraging blood‐based and digital biomarkers starts with understanding the current biomarker landscape and considering opportunities for widespread implementation in primary care clinical practice.

**Methods:**

A multidisciplinary team representing neurology, neuropsychology, geriatrics, primary care, epidemiology, laboratory programs, and patient advocacy was convened to review a summary of current biomarker research findings and discuss barriers and opportunities to implement biomarkers as part of an AD consensus‐driven clinical care pathway.

**Results:**

The emergence of biomarkers has shifted diagnosis from primarily clinical to a biological definition of AD. However, there is currently no consensus on where biomarkers fit within an AD care pathway and when they should be utilized in primary care or dementia specialist care settings.

We found a relative paucity of published data on biomarker test accuracy in diagnosis outside tightly controlled research settings, limiting guidance around how results should be interpreted and managed in real‐world care settings. Evidence gaps are especially pressing for heterogeneous, diverse populations under‐represented in AD research.

New biomedical therapies specific to the pathobiology of AD are driving research on blood and digital biomarkers to inform optimal ways to accelerate identification. As most individuals with AD are not evaluated by specialists, accurate and usable information about the place of biomarkers in the diagnosis and treatment of cognitive impairment must reach primary care

**Conclusions:**

With growing interest in the promise of non‐invasive biomarkers to improve detection, differentiation, and diagnosis of AD, new research is needed to generate real‐world evidence about their performance across populations, how to interpret results, and how best to use them in patient management. Effective educational strategies are needed to disseminate high‐quality evidence that engages primary care and healthcare delivery systems in implementing optimal clinical pathways. More detailed learnings for successful care pathway implementation will be shared.

